# Preplanned safety analysis of the JFMC37-0801 trial: a randomized phase III study of six months versus twelve months of capecitabine as adjuvant chemotherapy for stage III colon cancer

**DOI:** 10.1007/s10147-016-1083-9

**Published:** 2017-01-11

**Authors:** Takeshi Suto, Megumi Ishiguro, Chikuma Hamada, Katsuyuki Kunieda, Hiroyuki Masuko, Ken Kondo, Hideyuki Ishida, Genichi Nishimura, Kazuaki Sasaki, Takayuki Morita, Shoichi Hazama, Koutarou Maeda, Hideyuki Mishima, Hideyuki Ike, Sotaro Sadahiro, Kenichi Sugihara, Masazumi Okajima, Shigetoyo Saji, Junichi Sakamoto, Naohiro Tomita

**Affiliations:** 1 Department of Gastroenterological Surgery, Yamagata Prefectural Central Hospital, 1800 Aoyagi, Yamagata-shi, Yamagata 990-2214 Japan; 2 Department of Translational Oncology, Tokyo Medical and Dental University, 1-5-45 Yushima, Bunkyo-ku, Tokyo, 113-8519 Japan; 3 Graduate School of Engineering, Tokyo University of Science, 6-3-1 Niijuku, Katsushika-ku, Tokyo, 125-8585 Japan; 4 Department of Surgery, Gifu Prefectural General Medical Center, 4-6-1 Noishiki, Gifu-shi, Gifu 500-8717 Japan; 5 Department of Surgery, Nikko Memorial Hospital, 1-5-13 Shintomi-cho, Muroran-shi, Hokkaido 051-8501 Japan; 6 Department of Surgery, National Hospital Organization Nagoya Medical Center, 4-1-1 Sannomaru, Naka-ku, Nagoya-shi, Aichi 460-0001 Japan; 7 Department of Digestive Tract and General Surgery, Saitama Medical Center, Saitama Medical University, 1981 Kamoda, Kawagoe-shi, Saitama 350-8550 Japan; 8 Department of Surgery, Kanazawa Red Cross Hospital, 2-251 Mimma, Kanazawa-shi, Ishikawa 921-8162 Japan; 9 Department of Surgery, Otaru Ekisaikai Hospital, 1-10-17 Ironai, Otaru-shi, Hokkaido 047-0031 Japan; 10 Department of Surgery, Aomori Prefectural Central Hospital, 2-1-1 Higashitsukurimichi, Aomori-shi, Aomori 030-8553 Japan; 11 Department of Digestive Surgery and Surgical Oncology, Yamaguchi University Graduate School of Medicine, 1-1-1 Minamikogushi, Ube-shi, Yamaguchi 755-8505 Japan; 12 Department of Lower Gastrointestinal Surgery, Fujita Health University, 1-98 Dengakugakubo, Kutsukake-cho, Toyoake-shi, Aichi 470-1192 Japan; 13 Cancer Center, Aichi Medical University, 1-1 Yazakokarimata, Nagakute-shi, Aichi 480-1195 Japan; 14 Department of Surgery, Saiseikai Yokohama Southern Hospital, 3-2-10 Konandai, Konan-ku, Yokohama-shi, Kanagawa 234-8503 Japan; 15 Department of Surgery, Tokai University School of Medicine, 143 Shimokasuya, Isehara-shi, Kanagawa 259-1193 Japan; 16 Tokyo Medical and Dental University, 1-5-45 Yushima, Bunkyo-ku, Tokyo, 113-8519 Japan; 17 The Second Department of Surgery, Hiroshima University School of Medicine, 7-33 Motomachi, Naka-ku, Hiroshima-shi, Hiroshima 730-8518 Japan; 18 Japanese Foundation for Multidisciplinary Treatment of Cancer, 1-28-6 Kameido, Koto-ku, Tokyo, 136-0071 Japan; 19 Division of Lower Gastrointestinal Surgery, Department of Surgery, Hyogo College of Medicine, 1-1 Mukogawa-cho, Nishinomiya-shi, Hyogo 663-8501 Japan

**Keywords:** Colon cancer, Adjuvant chemotherapy, Capecitabine, Treatment duration, Adverse events, Hand-foot syndrome

## Abstract

**Background:**

Six months of adjuvant chemotherapy is regarded as the standard of care for patients with stage III colon cancer. However, whether longer treatment can improve prognosis has not been fully investigated. We conducted a phase III study comparing 6 and 12 months of adjuvant capecitabine chemotherapy for stage III colon cancer, and report here the results of our preplanned safety analysis.

**Methods:**

Patients aged 20–79 years with curatively resected stage III colon cancer were randomly assigned to receive 8 cycles (6 months) or 16 cycles (12 months) of capecitabine (2500 mg/m^2^/day on days 1–14 of each 21-day cycle). Treatment exposure and adverse events (AEs) were evaluated.

**Results:**

A total of 1304 patients (642 and 636 in the 6-month and 12-month groups, respectively) were analyzed. The most common AE was hand-foot syndrome (HFS). HFS, leukocytopenia, neutropenia, and hyperbilirubinemia (any grade) occurred more frequently in the 12-month group than in the 6-month group. HFS was the only grade ≥3 AE to have a significantly higher incidence in the 12-month group (23 vs 17%, *p* = 0.011). The completion rate for 8 cycles was 72% in both groups, while that for 16 cycles was 46% in the 12-month group. HFS was the most common AE requiring dose reduction and treatment discontinuation.

**Conclusions:**

Twelve months of adjuvant capecitabine demonstrated a higher cumulative incidence of HFS compared to the standard 6-month treatment period, while toxicities after 12 months of capecitabine were clinically acceptable.

**Trial registration:**

UMIN-CTR, UMIN000001367.

## Introduction

Colorectal cancer (CRC) is one of the most common cancers in Japan, with over 147,000 new cases expected in 2016 [1]. Postoperative adjuvant chemotherapy for patients with stage III CRC is the internationally accepted standard of care to improve patient survival.

In the mid-1990s, based on the results of several studies [2, 3], a 6-month course of intravenous 5-fluorouracil (5-FU) plus leucovorin (LV) came to be regarded as the standard regimen of adjuvant chemotherapy for colon cancer. From the following studies investigating oral FUs (such as tegafur-uracil [UFT] plus LV, capecitabine), and oxaliplatin-containing regimens (i.e., FOLFOX and CapeOX) as adjuvant chemotherapy for colon cancer, Western countries selected 6 months as the standard treatment duration [4–7]. Therefore, 6 months of adjuvant chemotherapy has been recognized as the global clinical standard.

On the other hand, by analyzing the data of >20,800 patients from 18 randomized controlled studies (RCTs) in the Adjuvant Colon Cancer Endpoints (ACCENT) database, Sargent et al. [8] demonstrated that the risk of stage II-III CRC recurrence was highest between 12 and 18 months after surgery and proposed that decreasing the recurrence risk at 12–18 months after surgery might improve survival. The results of a meta-analysis of three studies (JFMC 7-1, 7-2, and 15) by Hamada et al. [9], investigating the risk of recurrence in 2848 patients with curatively resected colon cancer followed by 1-year administration of oral FUs, strongly suggested that 12 months of oral FU drugs might translate the short-term (1–2 years) reduction in the risk of recurrence into a delayed advantage in overall survival (OS).

Those findings suggest that extending adjuvant oral FU therapy from 6 to 12 months might be able to improve prognosis. However, whether 12 months of adjuvant chemotherapy can decrease the peak of recurrence risk between 12 and 18 months postoperatively and improve survival has not been investigated by RCT. Therefore, we conducted a phase III study, JFMC37-0801 (UMIN-CTR; UMIN000001367), to compare 6 and 12 months of adjuvant chemotherapy using capecitabine (Chugai Pharmaceutical Co. Ltd., Tokyo, Japan), the most commonly used oral FU for CRC worldwide, in patients with stage III colon cancer.

The safety of adjuvant capecitabine for colon cancer has not been studied in a large sample of Japanese patients, even though adjuvant capecitabine is widely used clinically for CRC in Japan. Furthermore, it is unclear whether a longer treatment period might influence the incidence and severity of adverse events (AEs). We therefore report the results of a preplanned safety analysis, to increase the safety of capecitabine use in clinical practice.

## Patients and methods

### Enrollment and assignment

This study was conducted in accordance with the Declaration of Helsinki and the Ethical Guidelines for Clinical Research in Japan, and was approved by the Institutional Review Boards of each participating institute. Written informed consent was obtained from all patients before enrollment, and eligible patients were centrally registered.

The main eligibility criteria were (1) histologically confirmed stage III colon adenocarcinoma; (2) curatively resected with extended lymph node dissection (D2 or D3 in the Japanese Classification of Colorectal Carcinoma, 8th edition) [10]; (3) aged 20–79 years; (4) Eastern Cooperative Oncology Group performance status (ECOG-PS) of 0 to 1; (5) no prior chemotherapy or radiotherapy for CRC; (6) no other active malignancies; (7) adequate oral intake; (8) preserved major organ functions, and (9) no uncontrollable severe infection.

### Randomization and masking

After confirming eligibility, enrolled patients were randomly assigned to receive either 8 cycles (6 months) or 16 cycles (12 months) of capecitabine at the central registration center, using a minimization method, with stratification by lymph node metastasis (N1 or N2-3 in the Japanese Classification of Colorectal Carcinoma, 8th edition) [10] and institution. The assigned treatment arm was not blinded from both investigators and patients.

### Protocol treatment

Capecitabine was orally given at a dose of 1250 mg/m^2^ twice daily after meals for 14 consecutive days, followed by a 7-day rest. This 3-week treatment comprised 1 cycle. The control group (6M group) received 8 cycles and the study group (12M group) received 16 cycles. After completing the scheduled treatment, each group was switched to the follow-up schedule defined in the protocol, without any treatment until confirmation of metastasis or recurrence.

The assigned treatment was started within 8 weeks after surgery. During treatment, clinical findings and laboratory values were evaluated at least every 3 weeks. Evaluation at the beginning of each cycle was mandatory. Patients received treatment if they fulfilled the following criteria—leukocytes ≥3000/mm^3^, neutrophils ≥1500/mm^3^, platelets ≥75,000/mm^3^, aspartate aminotransferase (AST) and alanine aminotransferase (ALT) ≤2.5 × upper limit of normal (ULN), total bilirubin ≤1.5 × ULN, creatinine <1.5 × ULN, and no higher than grade 1 non-hematologic toxicities (i.e., anorexia, nausea, vomiting, and diarrhea). If the criteria for starting/continuing treatment were not fulfilled, treatment was postponed or temporarily suspended until AEs had improved sufficiently to meet the criteria. Supportive care including antiemetics, antidiarrheal drugs, liver supporting therapy (e.g., ursodeoxycholic acid), granulocyte colony-stimulating factor, oral vitamin B6, and external use of hydrating cream and steroids were allowed when physicians considered necessary.

Depending upon the severity of the AEs at the time of treatment suspension, the dose of capecitabine was reduced in accordance with the protocol. When a grade 2 AE developed the first time, treatment with capecitabine was suspended until the AE improved to grade ≤1, and then resumed at the same dose. If a grade 2 AE occurred twice or if a grade 3 AE occurred, the dose of capecitabine was reduced by 25%. The minimum dose was 50% of the initial dose recommended in the protocol.

The treatment was discontinued if (1) recurrence or other malignancies developed; (2) a grade 4 AE occurred; (3) treatment could not be resumed within 21 days after its postponement or temporary suspension; (4) further dose reduction was necessary even after the specified dose was reduced by two levels (−50%); (5) the physician judged that the protocol treatment was too difficult to continue; (6) the patient requested discontinuation of the treatment, and (7) the patient withdrew their informed consent.

### Data collection

Treatment information, such as the daily dose and the number of days of administration in each cycle, was collected from the case report forms of each patient. The relative dose intensity (RDI) for each cycle was defined as the ratio of the actual cumulative dose to the protocol-specified cumulative dose in each cycle. Completion rate of the protocol treatment was defined as the ratio of the number of patients who completed 8 or 16 cycles of capecitabine treatment to the number of patients included the safety analysis set of each treatment group.

The type and severity of AEs in each cycle were evaluated according to the National Cancer Institute Common Terminology Criteria for Adverse Events version 3.0. The most severe grade of each AE during each cycle was reported.

### Statistical analysis

All statistical analyses were performed using SAS software version 9.2 (SAS Institute, Cary, NC, USA). Descriptive statistics such as means, standard deviations, and medians were calculated. The chi-squared test was used to compare the incidence of AEs between the treatment groups. A *p* value <0.05 was considered significant.

## Results

### Patient characteristics

**Fig. 1:**
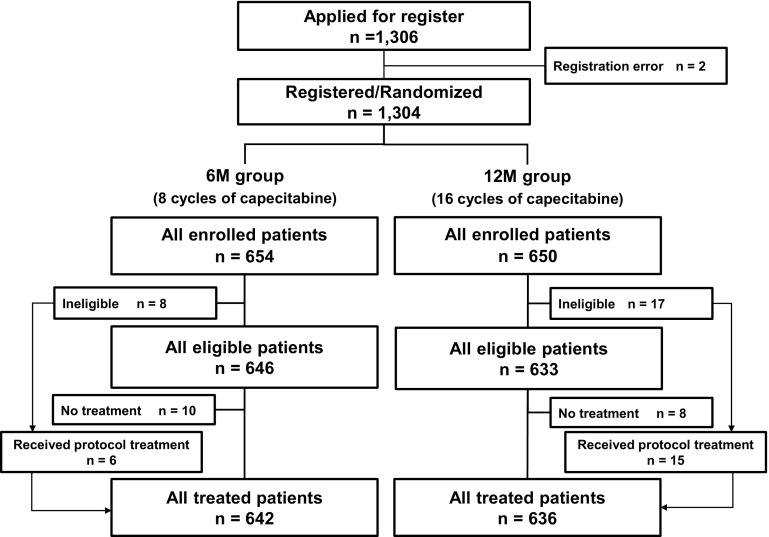
CONSORT diagram

From September 2008 through December 2009, a total of 1304 patients were enrolled from 333 institutes in Japan, and randomized. Of these, 1278 patients (642 in the 6M group and 636 in the 12M group) who received capecitabine treatment were included in the safety analysis set **(**Fig. 1). All data for the analysis were finalized in March of 2016.

Patient characteristics are shown in Table 1. Median age at enrollment was 65 years (range 23–79); 53.7% were male, and 96.1% were PS 0. Baseline characteristics of the two treatment groups were well balanced.

**Table 1:** Patient characteristics

	6M group (*n* = 642)		12M group (*n* = 636)	
	*n*	(%)	*n*	(%)
Age				
Median [range]	65 [23–79]		65 [34–79]	
Gender				
Male	347	(54.0%)	339	(53.3%)
Female	295	(46.0%)	297	(46.7%)
ECOG performance status				
0	611	(95.2%)	617	(97.0%)
1	31	(4.8%)	19	(3.0%)
Creatinine clearance (mL/min)				
Median [range]	76.6 [35.1–350.4]		78.1 [39.2–176.3]	
Body surface area (m^2^)				
Median [range]	1.59 [1.06–2.42]		1.56 [1.10–2.14]	
<1.33 (dose 3000 mg/body/day)	41	(6.4%)	48	(7.5%)
≥1.33 to <1.57 (dose 3600 mg/body/day)	262	(40.8%)	272	(42.8%)
≥1.57 to <1.81 (dose 4200 mg/body/day)	274	(42.7%)	261	(41.0%)
≥1.81 (dose 4800 mg/body/day)	65	(10.1%)	55	(8.6%)
Tumor location				
Right-sided colon (C, A, T)	261	(40.6%)	258	(40.6%)
Left-sided colon (D, S)	247	(38.5%)	247	(38.8%)
Rectosigmoid colon	134	(20.9%)	131	(20.6%)
Depth of tumor invasion (TNM 7th)				
T1	44	(6.9%)	45	(7.1%)
T2	54	(8.4%)	52	(8.2%)
T3	360	(56.1%)	355	(55.8%)
T4	184	(28.7%)	184	(28.9%)
LN metastasis (JSCCR classification^a^)				
N1	491	(76.5%)	486	(76.4%)
N2	120	(18.7%)	121	(19.0%)
N3	31	(4.8%)	29	(4.6%)
Stage (TNM 7th)				
IIIA	91	(14.2%)	93	(14.6%)
IIIB	455	(70.9%)	450	(70.8%)
IIIC	96	(15.0%)	93	(14.6%)
Scope of LN dissection (JSCCR classification^a^)				
D2	129	(20.1%)	133	(20.9%)
D3	513	(79.9%)	503	(79.1%)
Surgical approach				
Open (conventional)	371	(57.8%)	388	(61.0%)
Laparoscopic	271	(42.2%)	248	(39.0%)

*ECOG* Eastern Cooperative Oncology Group, *C* cecum, *A* ascending colon, *T* transverse colon, *D* descending colon, *S* sigmoid colon, *LN* lymph node

N1: Metastasis in 1–3 pericolic/perirectal LNs or intermediate LNs (LNs along the colic artery)

N2: Metastasis in ≥4 pericolic/perirectal or intermediate LNs

N3: Metastasis in LNs around the origin of the ileocolic, right colic, middle colic, or inferior mesenteric artery

D2: Complete dissection of pericolic/perirectal and intermediate LNs

D3: Complete dissection of all regional LNs

^a^Defined in the Japanese Classification of Colorectal Carcinoma 8th edition, published by the Japanese Society for Cancer of the Colon and Rectum (JSCCR) [10]

### Treatment duration

The median number of administered cycles was 8 in the 6M group and 15 in the 12M group. The completion rate for 8 cycles of capecitabine was similar in the 6M group (71.5%) and 12M group (71.7%). The final 16-cycle completion rate in the 12M group was 46.1% (Table 2). In both treatment groups, treatment discontinuation occurred at a similar frequency during both the first and last 8 cycles. Among 417 patients in the 12M group who began the 9th cycle, 293 (70.3%) completed 16 cycles (Table 2).

**Table 2:** Treatment discontinuation by cycle

	6M group (*n* = 642)		12M group (*n* = 636)	
	*n*	(%)	*n*	(%)
No. of patients discontinued				
During cycle 1	23	(3.6%)	25	(3.9%)
During cycle 2	21	(3.3%)	32	(5.0%)
During cycle 3	37	(5.8%)	33	(5.2%)
During cycle 4	21	(3.3%)	26	(4.1%)
During cycle 5	23	(3.6%)	20	(3.1%)
During cycle 6	21	(3.3%)	13	(2.0%)
During cycle 7	23	(3.6%)	28	(4.4%)
During cycle 8	14	(2.2%)	42	(6.6%)
During cycle 9	–	–	19	(3.0%)
During cycle 10	–	–	15	(2.4%)
During cycle 11	–	–	16	(2.5%)
During cycle 12	–	–	10	(1.6%)
During cycle 13	–	–	14	(2.2%)
During cycle 14	–	–	14	(2.2%)
During cycle 15	–	–	25	(3.9%)
During cycle 16	–	–	11	(1.7%)
No. of patients completed 8 cycles of capecitabine	459	(71.5%)	456	(71.7%)
No. of patients completed 16 cycles of capecitabine	–	–	293	(46.1%)

### Reasons for treatment discontinuation

In all, 183 patients (28.5%) and 343 patients (53.9%) in the 6M group and 12M group, respectively, dropped out from the protocol treatment. Their reasons for treatment discontinuation are listed in Table 3. The distribution of patients according to reason for discontinuation was similar between the two groups. AEs (listed in the protocol as treatment discontinuation criteria) or physician’s judgment were the most common reasons for discontinuation, and occurred in 50.3, 53.0, and 34.7% of discontinued patients during cycles 1–8 in the 6M group, and cycles 1–8 and 9–16 in the 12M group, respectively. Approximately 14.8, 17.4, and 15.3% of discontinued patients, respectively, requested to discontinue the treatment because of AEs not mentioned in the discontinuation criteria.

**Table 3:** Reasons for treatment discontinuation

	6M group (*n* = 642)		12M group (*n* = 636)			
	*n*	(%)	During cycles 1–8		During cycles 9–16	
			*n*	(%)	*n*	(%)
No. of patients with discontinuation	183	(100%)	219	(100%)	124	(100%)
*Reasons for discontinuation*						
Oncologic events						
Recurrences	17	(9.3%)	12	(5.5%)	12	(9.7%)
Second cancers	3	(1.6%)	2	(0.9%)	1	(0.8%)
Adverse events (AEs)						
AEs (listed on the discontinuation criteria) or physician’s judgement	92	(50.3%)	116	(53.0%)	43	(34.7%)
Patient’s request due to AEs not mentioned in the discontinuation criteria	27	(14.8%)	38	(17.4%)	19	(15.3%)
Others						
Aggravation of comorbidities	5	(2.7%)	9	(4.1%)	8	(6.5%)
Patient’s request due to non-medical reasons	16	(8.7%)	35	(16.0%)	20	(16.1%)
Others	23	(12.6%)	7	(3.2%)	21	(16.9%)

Types of AEs leading to treatment discontinuation are presented in Table 4. Distribution of patients according to cause of discontinuation was similar between the 6M and 12M group, and between cycles 1–8 and cycles 9–16 in the 12M group. HFS was the most common discontinuation criteria-specified AE leading to discontinuation or the basis for physician's judgment to discontinue treatment. The proportion of patients requesting treatment discontinuation because of HFS was similar to that requesting treatment discontinuation because of other non-hematologic toxicities. Overall, HFS was the leading AE for treatment discontinuation.

**Table 4:** Adverse events causing discontinuation of treatment

		6M group (*n* = 642)	12M group (*n* = 636)	
			During cycles 1–8	During cycles 9–16
Discontinuation due to AEs		119 patients 123 AEs (100%)	154 patients 160 AEs (100%)	62 patients 65 AEs (100%)
AEs (listed on the discontinuation criteria) or physician’s judgement	Hematologic toxicities	18 (14.6%)	25 (15.6%)	9 (13.8%)
	Abnormal liver function	18 (14.6%)	22 (15.8%)	8 (12.3%)
	Hand-foot syndrome	38 (30.9%)	60 (37.5%)	19 (29.2%)
	Non-hematologic toxicities^a^	18 (14.6%)	11 (6.9%)	7 (10.8%)
Patient’s request due to AEs not mentioned in the discontinuation criteria	Hematologic toxicities	2 (1.6%)	2 (1.3%)	1 (1.5%)
	Abnormal liver function	0 (0%)	0 (0%)	0 (0%)
	Hand-foot syndrome	11 (8.9%)	21 (13.1%)	10 (15.4%)
	Non-hematologic toxicities*	18 (14.6%)	18 (11.3%)	9 (13.8%)
	Unknown	0 (0%)	1 (0.6%)	2 (3.1%)

*AEs* adverse events

^a^Not including hand-foot syndrome

### Dose modification

The dose was reduced 314 times in 241 patients (37.5%) in the 6M group and 477 times in 306 patients (48.1%) in the 12M group. In the 12M group, the proportion of patients with dose reduction was lower during cycles 9–16 than during cycles 1–8 (26.1 vs 40.4%) (Table 5). The most common reason for dose reduction was HFS, which occurred 191 times (60.8%) in the 6M group and 290 times (60.8%) in the 12M group.

**Table 5:** Dose reduction

Dose reduction	6M group (*n* = 642)	12M group (*n* = 636)		
		Overall (*n* = 636)	During cycles 1–8 (*n* = 636)	During cycles 9–16 (*n* = 417)
	*n* (%)	*n* (%)	*n* (%)	*n* (%)
(−)	401 (62.5%)	330 (51.9%)	379 (59.6%)	308 (73.9%)
(+)	241 (37.5%)	306 (48.1%)	257 (40.4%)	109 (26.1%)

**Fig. 2:**
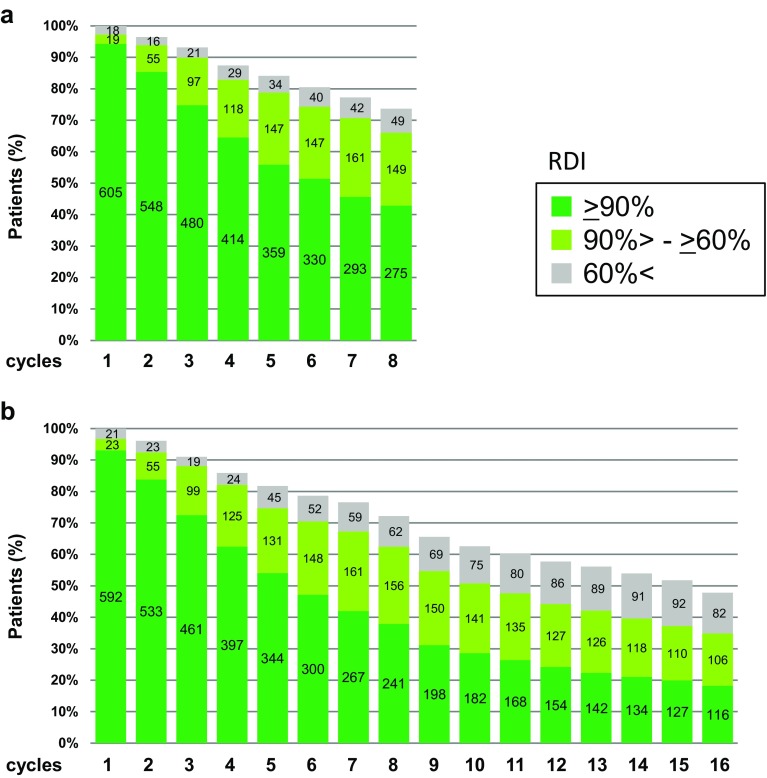
Relative dose intensity in each cycle. **a** 6M group; **b** 12M group

The RDI for each cycle is shown in Fig. 2. RDI decreased gradually with each successive treatment cycle, and was ≥60% in 424 patients (66.0%) in the 6M group at cycle 8, 397 patients (62.4%) in the 12M group at cycle 8, and 222 patients (34.9%) in the 12M group at cycle 16. The mean RDI for the entire treatment period and all patients, including those who discontinued prematurely, was 79.5% in the 6M group and 61.3% in the 12M group (median was 89.6 and 65.4%, respectively).

### Safety profile (6M group vs 12M group)

A total of 589 patients (91.7%) in the 6M group and 602 patients (94.7%) in the 12M group experienced AEs (*p* = 0.051). Moreover, 158 patients (24.6%) in the 6M group and 197 patients (31.0%) in the 12M group experienced grade ≥3 AEs (*p* = 0.013). The incidence of major AEs (by worst grade throughout the treatment period) is shown in Table 6. The most common AE was HFS; the incidence of grade ≥3 HFS was 16.8 and 22.6% in the 6M group and 12M group, respectively. Other grade ≥3 AEs with ≥1% incidence included neutropenia, diarrhea, fatigue, and anorexia. There was no treatment-related death in the study.

**Table 6:** Incidence of adverse events by the treatment group

	6M group (*n* = 642)		12M group (*n* = 636)		Any grade, *p* value^a^
	Any grade (%)	Grade ≥3 (%)	Any grade (%)	Grade ≥3 (%)	
Hemoglobin	36.0	0.3	40.6	0.3	0.103
Leukocytes	19.2	0.6	25.6	0.3	0.007
Neutrophils	15.4	2.6	20.6	3.6	0.020
Platelets	13.7	0.5	13.7	0.5	0.839
Total bilirubin	31.2	0.5	39.2	0.8	0.003
AST	18.1	0.2	21.7	0.6	0.120
ALT	17.0	0.3	20.6	0.5	0.113
Creatinine	5.5	0	8.0	0	0.085
Anorexia	20.9	1.2	21.4	0.9	0.877
Nausea	15.6	0.5	13.7	0.6	0.379
Vomiting	6.4	0.2	5.2	0.5	0.425
Stomatitis	17.9	0.8	21.9	0.9	0.090
Diarrhea	18.1	3.0	14.9	2.0	0.152
Fatigue	15.3	1.7	16.0	1.3	0.762
Rash	10.9	0.5	10.1	0.2	0.690
Hyperpigmentation	27.9	0	25.2	0.5	0.299
Alopecia	1.9	0.2	2.5	0.2	0.550
Hand-foot syndrome	72.0	16.8	77.0	22.6	0.043

*AST* aspartate aminotransferase, *ALT* alanine aminotransferase

^a^chi-squared test

AEs (any grade) with a higher incidence in the 12M group than in the 6M group included leukocytopenia (25.6 vs 19.2%, *p* = 0.007), neutropenia (20.6 vs 15.4%, *p* = 0.020), hyperbilirubinemia (39.2 vs 31.2%, *p* = 0.003), and HFS (77.0 vs 72.0%, *p* = 0.043). HFS was the only grade ≥3 AE to occur more frequently in the 12M group than in the 6M group (22.6 vs 16.8%, *p* = 0.011).

### Safety profile (cycles 1–8 vs cycles 9–16)

A comparison of the incidence of AEs between the first 8 cycles and the second 8 cycles in the 12M group (Table 7) revealed no difference in the incidence of hematologic toxicities. Another non-hematological AEs (any grade) including anorexia (9.1 vs 18.4%, *p* < 0.001), nausea (4.1 vs 12.4%, *p* < 0.001), stomatitis (10.6 vs 18.9%, *p* < 0.001), diarrhea (6.7 vs 11.6%, *p* = 0.011), fatigue (7.9 vs 13.1%, *p* = 0.012), and vomiting (1.7 vs 4.2%, *p* = 0.034) were lower in cycles 9–16 than in cycles 1–8. This shows that the incidence of gastrointestinal toxicities was lower during the later period of treatment while that of hematologic toxicities remained fairly constant over the entire treatment course. HFS was the only grade ≥3 AE with a significantly lower incidence in the later period (8.6 vs 19.0%, *p* < 0.001). However, the incidence of grade ≥3 HFS (8.6%) was by far the highest of any grade ≥3 AE occurring during cycles 9–16.

**Table 7:** Adverse events in the 12M group during cycles 1–8 and 9–16

	During cycles 1–8 (*n* = 636)		During cycles 9–16 (*n* = 417)	
	Any grade (%)	Grade ≥3 (%)	Any grade (%)	Grade ≥3 (%)
Hemoglobin	34.9	0	31.7	0.5
Leukocytes	19.3	0.3	21.1	0
Neutrophils	17.6	2.8	12.9	2.2
Platelets	9.6	0.5	12.7	0
Total bilirubin	32.5	0.6	36.0	0.5
AST	16.2	0.3	17.5	0.5
ALT	15.4	0.3	14.1	0.2
Creatinine	5.2	0	7.7	0
Anorexia	18.4	0.9	9.1	0
Nausea	12.4	0.6	4.1	0
Vomiting	4.2	0.5	1.7	0
Stomatitis	18.9	0.9	10.6	0
Diarrhea	11.6	1.7	6.7	0.5
Fatigue	13.1	0.9	7.9	0.5
Rash	7.5	0.2	6.7	0
Hyperpigmentation	20.8	0.5	18.7	0
Alopecia	1.9	0	1.4	0.2
Hand-foot syndrome	71.1	19.0	66.7	8.6

*AST* aspartate aminotransferase, *ALT* alanine aminotransferase

**Fig. 3:**
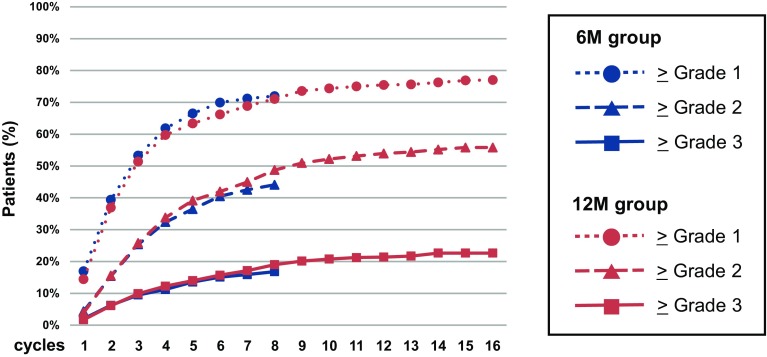
Cumulative incidence of hand-foot syndrome

The cumulative incidence of grade ≥1, grade ≥2, and grade ≥3 HFS by treatment group is shown in Fig. 3. The rise in cumulative onset of HFS during cycles 1–8 was quite similar between the 6M group and 12M group. In the 12M group, the rise in cumulative onset was gradual and constant even during cycles 9–16.

## Discussion

We compared the treatment details and AE profile after 6 and 12 months of adjuvant capecitabine in 1278 Japanese patients with stage III colon cancer. This is the first prospective RCT data demonstrating the safety of adjuvant capecitabine in a large sample of Japanese patients.

The most common AE was HFS, a characteristic toxic reaction to capecitabine; 72.0 and 77.0% of patients experienced grade ≥1 HFS, and 16.8 and 22.6% experienced grade ≥3 HFS in the 6M group and 12M group, respectively. The incidences of other AEs were relatively low overall and acceptable.

A comparison of the AE profile between our study and the X-ACT trial [5], a pivotal study of adjuvant capecitabine for colon cancer patients (Table 8), found no difference in the incidence of grade ≥3 HFS between Japanese and Western patients. It was reported that oral FUs were less frequently associated with gastrointestinal toxicities in Asian patients than in Caucasian patients [11, 12]. Indeed, the incidences of gastrointestinal toxicities (i.e., diarrhea, nausea, vomiting, and stomatitis) were lower in our study than in the X-ACT. When compared to toxicities associated with other oral FUs (such as UFT/LV and S-1) used as adjuvant chemotherapy for CRC in Japan **(**Table 8), anorexia, nausea, and diarrhea associated with capecitabine were less frequent [11, 12]. From these findings, we suggest that capecitabine might be an easy-to-use oral FU for Japanese patients, when HFS is well-controlled.

**Table 8:**
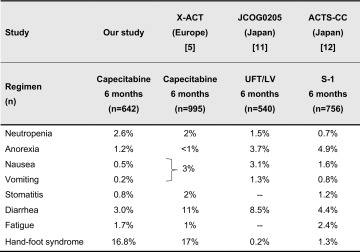
Reported incidence of grade ≥3 adverse events in other studies [5, 11, 12]

HFS was the only grade ≥3 AE whose incidence increased with treatment during the period extending from 6 to 12 months. Although lower in the second 6-month period than in the first 6-month period, the overall incidence of HFS increased gradually and constantly even in the later period and therefore was higher in the 12M group than in the 6M group (Table 6).

Although the completion rate for 6-month treatment in our study was similar to that in other studies of adjuvant oral FU therapy for colon cancer [4, 5, 11–13], the completion rate for 12-month treatment was <50%. Approximately half of the discontinuations during cycles 9–16 was due to AEs, most commonly HFS. These findings indicate that prolonging the treatment duration resulted in a higher incidence of HFS with constant cumulative onset, and that HFS led mainly to dose reduction and treatment discontinuation, but was not lethal. Therefore, effective management of HFS could improve the completion rate for 12-month treatment.

The use of a hydrating cream, external steroid, and oral vitamin B6 is the accepted supportive treatment for HFS [14] and was allowed in our study. However, in this study, 477 (74.3%) and 471 (74.5%) patients in the 6M group and 12M group, respectively, used oral vitamin B6. These recommended supportive measures should be taken in all cases, even at the start of the treatment.

The X-ACT trial reported that appropriate dose reduction does not impair the efficacy of adjuvant capecitabine therapy and that the survival rate was better among those who developed HFS than among those who did not [15]. These observations suggest that appropriate dose reduction to manage HFS is important to maintain the treatment duration and to improve patient outcome. The result of the primary object of our study, a comparison of survival rate between the 6M group and 12M group, will be available in late 2016.

In conclusion, compared to the standard 6 month-treatment, the cumulative incidence of HFS (the most common reason for treatment dropout) increased further after 12 months of adjuvant capecitabine, although overall, the incidence and severity of AEs after 12 months of capecitabine were acceptable. Appropriate dose modification and supportive care could reduce the rate of treatment discontinuation due to HFS and improve treatment compliance.
